# Editorial: Unravelling the wildlife gut microbiome: the crucial role of gut microbiomes in wildlife conservation strategies

**DOI:** 10.3389/fmicb.2026.1838495

**Published:** 2026-04-21

**Authors:** Houqiang Luo, Zhangzhou Shen, Yongli Jian, Meng Wang, Shuai Luo, Juan Wang, Li Nan, Li Tang, Mujeeb Ur Rehman, Franck Carbonero

**Affiliations:** 1 College of Animal Science, Wenzhou Vocational College of Science and Technology, Wenzhou, Zhejiang, China; 2 Medical School, Hubei Polytechnic University, Huangshi, Hubei, China; 3 College of Veterinary Medicine, Sichuan Agricultural University, Chengdu, Sichuan, China; 4 Directorate Planning and Development, Livestock and Dairy Development Department, Quetta, Balochistan, Pakistan; 5 Department of Nutrition and Exercise Physiology, Elson Floyd College of Medicine, Washington State University, Spokane, WA, United States

**Keywords:** fecal microbiota transplantation in conservation, gut health, gut microbiota and biodiversity, wildlife disease and microbiomes, wildlife gut microbiome

In recent years, researchers have increasingly recognized the importance of the gut microbiota in promoting intestinal inflammation and maintaining host health in wildlife. Those diverse communities of microorganisms, including bacteria, archaea, fungi, and viruses, living in the intestine of wild animals are referred to collectively as the intestinal microbiota, which play a significant role in digestion, immune function and several physiological processes (Liu et al., 2022; Tyagi, 2024; Zhang et al.). The gut microbiome is the wildlife's second genome, which facilitates the co-evolution with the host to establish a sophisticated and resilient microecosystem in the realm of vertebrate biology (Jiang et al., 2026). The microbiome profoundly influences host health, nutrition, and resilience, influencing survival and adaptability. Understanding the intricate relationships among microbial groups and with their animal hosts is essential for understanding fundamental biological and ecological processes, and to implement successful conservation strategies (Carrillo et al., 2024; Ullah et al., 2024).

The aim of the Frontiers in Microbiology Research Topic “*Unravelling the wildlife gut microbiome: the crucial role of gut microbiomes in wildlife conservation strategies*” was to collate state-of-the-art articles in the field related to the dynamic interactions within wildlife gut microbiomes to expand scientific understanding by integrating microbiome research with wildlife conservation efforts to devise and refine strategies, and to enhance survival and ecosystem health.

This Research Topic in Frontiers in Microbiology, titled “*Unravelling the wildlife gut microbiome: the crucial role of gut microbiomes in wildlife conservation strategies*,” aimed to bring together state-of-the-art articles on dynamic interactions within wildlife gut microbiomes. By integrating microbiome research with conservation efforts, the aim was to expand scientific understanding, develop and refine conservation strategies, and ultimately improve wildlife survival and ecosystem health. The 23 original articles and two summary articles gathered in this Research Topic have expanded our knowledge of the role of the wildlife microbiome.

Many external factors, including seasonal shifts, dietary changes, social density, and environmental pollutants, drive dynamic changes in the gut microbial communities of wildlife. Schweikhard et al. observed that variations in the seasonal diet of captive Coquerel's sifakas lead to alterations in the makeup of their fecal microbial populations, with notable differences between summer and winter samples. Although these compositional changes occurred, the general diversity of the intestinal microbiota stayed consistent when frozen foliage was supplied during the winter months. These findings indicate that dietary management can support the preservation of a stable microbial community framework, even when seasonal dietary modifications are required. Liu et al. reported coordinated seasonal shifts in both bacterial and fungal communities of wild Francois' langurs, with mucin-degrading Akkermansia dominating in summer and plant biomass-degrading Cercophora enriched in winter, highlighting complementary microbial strategies for seasonal dietary adaptation. Botsidou et al. revealed that skin, but not gut, microbial communities in Antarctic fur seals were sensitive to social density, with high-density colonies exhibiting lower skin microbial diversity and enrichment of pathogenic phyla, indicating that host-microbe interactions vary by body site and environmental exposure. Jiang et al. reported that the web-footed shrew maintains a stable, specialized diet of benthic macroinvertebrates and fish, with a gut microbiota dominated by Proteobacteria and enriched in genes related to fatty acid metabolism, illustrating how diet-microbiome coevolution facilitates semi-aquatic niche adaptation.

New microbiome insights can directly promote conservation practice and the development of non-invasive tools for population management. Zhai et al. demonstrated that a 20-day pre-release environmental acclimation protocol in Kaluga sturgeon optimizes digestive enzyme activity, immune function, and gut microbiota stability, providing a concrete framework to enhance post-stocking survival in hatchery-reared fish. Forehand et al. showed that while captivity alters the gut microbiome of head-started lizards, the microbiome rapidly converges to a wild-type state within 2 months post-release, suggesting that captive-induced microbial changes are transient and do not pose a long-term barrier to reintroduction success. The study on Amur tigers by Hu et al. introduced a novel non-invasive method that integrates age-associated gut microbial biomarkers with microsatellite-based kinship analysis to construct accurate family pedigrees. Their results demonstrate that this method could be used to the utility of microbiomics for informing conservation genetics and population management.

The dietary specialization, developmental transitions, and adaptation to extreme environments are important for exploring the co-evolutionary dynamics between hosts and their microbiomes. Jiang et al. provided a compelling case of diet-microbiome coevolution in the web-footed shrew, where a specialized carnivorous diet is supported by a gut microbiota with reduced carbohydrate-active enzymes and enhanced fatty acid metabolism, enabling niche specialization and semi-aquatic adaptation. A study on Mongolian wild asses by Wang J. et al. revealed a clear age-dependent shift from a Bacillota-dominated community in juveniles to a Bacteroidota-dominated community in adults, reflecting a co-evolutionary adaptation that supports a transition from milk to a high-fiber diet in an arid desert ecosystem. Pinos et al. investigated bee microbiomes along an elevation gradient and found that host identity was a stronger predictor of community composition than elevation, with distinct responses among bee tribes, underscoring the primacy of host-microbe coevolutionary history in structuring microbial communities. Lan et al. compared native Guizhou horses and imported Dutch Warmblood horses, revealing breed-specific gut microbial signatures that likely reflect distinct genetic backgrounds and long-term adaptations to local environments, suggesting a co-evolutionary link between host genetics and microbial community structure.

The composition of non-invasive sampling, longitudinal tracking, and multi-kingdom analysis is important for wildlife microbiome research. Schweikhard et al. employed Oxford Nanopore sequencing technology to analyze the fecal microbial communities of all Coquerel's sifakas held in captivity across Europe. This study highlighted the practicality of conducting longitudinal sampling across the species' entire range and underscored the utility of high-throughput sequencing approaches for tracking and conserving populations of endangered species. Research conducted by Hu et al. on Amur tigers introduces an innovative, non-invasive technique that merges fecal microbiome profiling with microsatellite kinship assessment to estimate age and reconstruct family lineages. This approach establishes a robust methodological structure for incorporating microbiomic data into the field of wildlife conservation genetics. Karamendin et al. conducted a detailed characterization of post-mortem microbiome alterations in swans after lethal H_5_N_1_ infection, offering a thorough analysis of microbial dysbiosis in recently deceased wild birds. Their work underscores important methodological considerations for investigating pathogen-microbiome interactions in mortality events. Xiang et al. employed a dual-omics strategy, merging 16S rRNA gene sequencing with untargeted metabolomic profiling, to conduct a comprehensive comparative analysis of the gut microbial communities and metabolic landscapes across two distinct pangolin species. This approach underscores the utility of integrating taxonomic microbial composition with functional metabolic insights to enhance species-tailored health evaluations.

The intricate connections between gut microbiota and various disease conditions, ranging from infectious diseases to oxidative stress-related disorders, are vital to explore microbiome-targeted interventions for health management. Zhang et al. consolidated existing research on gut microbial imbalance as a pivotal factor in animal diarrhea, elucidating the processes through which advantageous microorganisms preserve intestinal balance and assessing microbiota-focused treatments as alternatives to antibiotics. Karamendin et al. uncovered that fatal H5N1 avian influenza in swans correlates with significant microbial disruption marked by a predominant presence of Campylobacter and Fusobacterium, indicating a widespread disturbance in the host-microbe balance that could exacerbate disease outcomes. Wang L. et al. established that *Bacillus subtilis* from yaks mitigates oxidative stress and liver damage induced by D-galactose in mice by adjusting the gut microbiota, enhancing microbial diversity, and triggering the Keap1/Nrf2 antioxidant mechanism, emphasizing the curative value of probiotics adapted to specific hosts. Qin et al. discovered a connection between microplastic ingestion, gut microbial disturbance, and diminished antioxidant function in broiler chickens, exposing a new route through which environmental contaminants affect host wellbeing by altering the microbiome. Research on Orinoco crocodiles by Castelli et al. indicated that *Helicobacter* spp. colonization is linked to decreased bacterial diversity and changes in community makeup, such as an increase in possible pathogens, highlighting how particular bacterial colonization influences microbial ecosystem dynamics and health in a highly threatened reptile species.

Overall, the articles in the present Research Topic collectively emphasize the importance of gut microbiota in promoting intestinal inflammation and maintaining host health benefits in wildlife. These studies shed light on novel therapeutic strategies for various health conditions, including animal diarrhea, gut microbial dysbiosis, oxidative stress, and liver injury. The findings underscore the significance of understanding the intricate relationships between these microbial communities and their animal hosts for developing innovative and effective therapeutic interventions in wildlife.

Recent research has focused on these interactions, revealing notable differences in the composition and functional roles of microbiomes among various species and habitats. However, there are still obstacles in our understanding of their broader ecological effects and their potential applications in conservation efforts. This strategy effectively addresses the escalating issue of antibiotic resistance and facilitates the advancement of personalized medical treatments. However, identifying the specific alternatives to antibiotics responsible for the observed effects presents a considerable challenge.

Taken together, the present Research Topic highlights the dynamic interactions of wildlife gut microbiomes with their environment and conservation contexts. It also introduces novel concepts for additional studies in this area, offering practical management approaches to improve wildlife conservation. Further investigation is needed to broaden scientific knowledge by combining microbiome studies with conservation initiatives, enabling the development and improvement of strategies that boost survival rates and ecosystem health.
